# 1615. Efficacy of Long-Acting Cabotegravir and Rilpivirine in a Diverse Group of Patients in a Real-World Setting

**DOI:** 10.1093/ofid/ofad500.1450

**Published:** 2023-11-27

**Authors:** Nicholas F Yared, Smitha Gudipati, Shannon Payne, Rija B R Alvi, John Cherian, John Di Lodivico, Norman Markowitz, Indira Brar

**Affiliations:** Henry Ford Health System, Detroit, Michigan; Henry Ford Health System, Detroit, Michigan; Henry Ford Health, Detroit, Michigan; Henry Ford Hospital, Detroit, Michigan; Henry Ford Health, Detroit, Michigan; Henry Ford Health, Detroit, Michigan; Henry Ford Health System, Detroit, Michigan; Henry Ford Hospital, Detroit, Michigan

## Abstract

**Background:**

Cabotegravir (CAB) + rilpivirine (RPV) is the first recommended complete long-acting (LA) regimen for maintenance of HIV-1 virologic suppression. The efficacy and safety of switching to CAB + RPV LA (CAR) has been shown in clinical trials. CAR injections offer less frequent dosing and address issues of adherence and disclosure related to daily oral cART. We describe the clinical characteristics and outcomes of switching a diverse group of people with HIV (PWH) to CAR in a real-world setting.

**Methods:**

A retrospective cohort study was performed to assess virologic efficacy of intramuscular CAR given every 4 or 8 weeks among adult PWH receiving care at Henry Ford Health ID Clinic by an interdisciplinary team of physicians, nurses, social workers, and a pharmacist. Efficacy was defined as HIV-1 RNA < 20 copies/mL at 3 months. Demographics, clinical characteristics, and outcomes were extracted from the electronic medical record.

**Results:**

We included the first 51 patients to receive CAR. Median age was 46 years (IQR 34 -59). Black individuals were 75%, cisgender males 84%, and transgender females 3.9% of participants. PWH were diagnosed a median of 12 years ago (IQR 11-17). At time of switch, 90% had HIV viral load (VL) < 20 copies/ml, and 9.8% of patients were viremic with < 75 copies/ml. Mean Cd4+ cell count was 871 cells/µL (IQR 632-1603). Prior to switch, 80% had received ≥ 2 cART regimens, 75% had INSTI exposure, and 45% had NNRTI exposure. Among 38 patients with HIV-1 genotypes available prior to switch, 4 had either baseline NNRTI or INSTI mutations (Table 2). For patients with VL data at 3 months, 37 of 38 (98%) had an undetectable VL. Virologic failure occurred in 1 PWH with BMI 35 who had a Y188L RT mutation in a 2009 genotype which did not include RPV (2011 approval), with subsequent emergence of pan-NNRTI and INSTI resistance.

**Figure:**
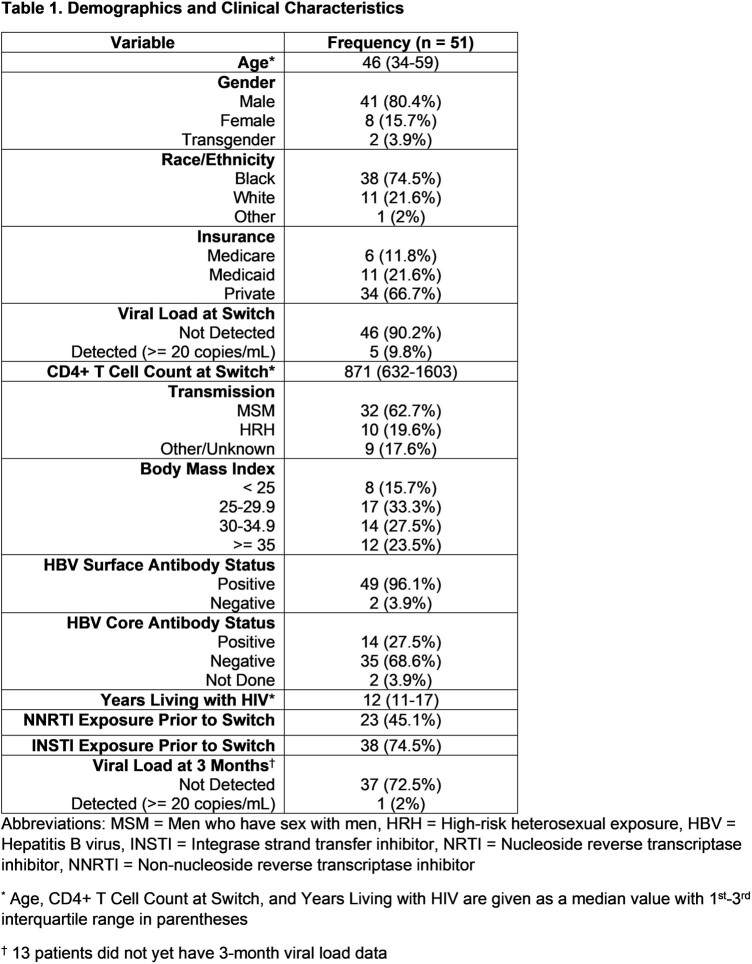

**Figure:**
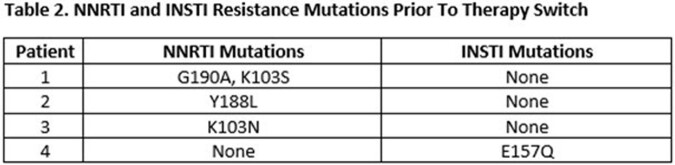

**Conclusion:**

A high degree of virologic suppression at 3 months was achieved among an older, diverse cohort of PWH cared for by an interdisciplinary team. Unrecognized baseline HIV resistance to NNRTI contributed to one virologic failure. It is important to assure that genotypic susceptibility interpretations are current and to carefully assess for eligibility before switching to CAR.

**Disclosures:**

**John Di Lodivico, PharmD**, Midwest AIDS Training & Education Center: Honoraria|ViiV Healthcare: Advisor/Consultant|Wayne State University: Honoraria **Indira Brar, MD**, Gilead: Advisor/Consultant|Gilead: Grant/Research Support|Gilead: Honoraria|Janssen: Grant/Research Support|Janssen: Honoraria|ViiV: Advisor/Consultant|ViiV: Grant/Research Support|ViiV: Honoraria

